# Differences in patterns of high-risk human papillomavirus infection between urban and rural low-resource settings: cross-sectional findings from Mali

**DOI:** 10.1186/1472-6874-13-4

**Published:** 2013-02-06

**Authors:** Nicholas H Schluterman, Samba O Sow, Cheick B Traore, Kamate Bakarou, Rokiatou Dembelé, Founé Sacko, Patti E Gravitt, J Kathleen Tracy

**Affiliations:** 1 Department of Epidemiology and Public Health, University of Maryland School of Medicine, Baltimore, MD, USA; 2 Centre Pour Le Developpement Des Vaccins, Bamako, Mali; 3 Institut National de Recherche en Santé Publique, Bamako, Mali; 4 Johns Hopkins Bloomberg School of Public Health, Baltimore, MD, USA

**Keywords:** Uterine cervical cancer, Reproductive health, HPV prevalence, Mali

## Abstract

**Background:**

The burden of cervical cancer is disproportionately high in low-resource settings. With limited implementation of human papillomavirus (HPV) vaccines on the horizon in the developing world, reliable data on the epidemiology of high-risk HPV (HR-HPV) infection in distinct geographic populations is essential to planners of vaccination programs. The purpose of this study was to determine whether urban patterns of HR-HPV occurrence can be generalized to rural areas of the same developing country, using data from Mali, West Africa, as an example.

**Methods:**

Urban and rural women in Mali participated in a structured interview and clinician exam, with collection of cervical samples for HPV DNA testing, to determine HR-HPV prevalence and correlates of infection. Correlates were assessed using bivariate analysis and logistic regression.

**Results:**

A total of 414 women (n=202 urban women; n=212 rural women) were recruited across both settings. The prevalence of HR-HPV infection in rural women was nearly twice that observed in urban women (23% v. 12%). Earlier age of sexual debut and fewer pregnancies were associated with HR-HPV infection among urban women, but not rural women. Twenty-six percent of urban women who had sexual intercourse by age 14 had an HR-HPV infection, compared to only 9% of those who had later sexual debut (p<0.01). Overall, age, income, and polygamy did not appear to have a relationship with HR-HPV infection.

**Conclusions:**

Compared to urban women, rural women were significantly more likely to be infected with high-risk HPV. The patterns and risk factors of HR-HPV infection may be different between geographic areas, even within the same developing country. The high prevalence in both groups suggests that nearly all rural women and most urban women in Mali will be infected with HR-HPV during their lifetime, so the effects of risk factors may not be statistically apparent. To control HPV and cervical cancer in West Africa and the rest of the developing world, planners should prioritize vaccination in high-burden areas.

## Background

The developing world bears a disproportionately high burden of cervical cancer [1]. Particularly affected is West Africa, where cervical cancer is the most common cause of cancer death among women. Mali, in West Africa, had an age-standardized incidence rate of the disease of 37.7 cases per 100,000 women in 2008, and a disease mortality rate of 28.4 deaths per 100,000 women—16 times the mortality rate seen in the US [1].

Human papillomavirus (HPV) is a common sexually-transmitted pathogen, of which several high-risk types are associated with nearly all cases of cervical cancer worldwide [2]. The high prevalence of HPV among women in Mali and elsewhere in West Africa [3,4] may be explained by risk factors including high parity and early age at sexual debut [5,6]. Lack of routine cervical screening and inadequate treatment options exacerbate the cervical cancer burden throughout that country and region [7-9].

HPV vaccination of adolescent girls may be on the horizon in the developing world. However, the drug and delivery costs of vaccination may prohibit widespread implementation in low-resource countries in the immediate future [10]. Donation or subsidization of the HPV vaccine by non-government organizations may provide a limited supply in sub-Saharan Africa in the near future [11]; countries will then need to reallocate their stretched public health resources to incorporate HPV vaccination into existing programs. To maximize the impact of this first wave of vaccination, researchers must first generate reliable country and population-specific epidemiological data for HPV and cervical cancer, demonstrating the subpopulations of greatest need.

Previous studies have examined correlates of HPV infection [9] and invasive cervical cancer [12] among urban women in Mali. A case-control study in Bamako-the capital and largest city in Mali-in the mid-1990s counted high parity and polygamy as risk factors for cervical cancer in that sample [12]. No rural epidemiological data on HPV or cervical cancer have yet been published from rural parts of Muslim West Africa, and estimates elsewhere in the developing world have relied heavily on urban data [13,14]. Furthermore, we are not aware of any published study yet that has used standardized instruments to compare patterns and correlates of HPV infection or cervical cancer between rural and urban areas within the same sub-Saharan African country. Existing urban data may not be generalizable to other areas even within the same country, where cultural practices, sanitation, health, behaviors, and sexual network construction may be far different.

The current study sought to determine whether the patterns of high-risk HPV (HR-HPV) infection are different between urban and rural areas of the same developing country, using pooled data from two cross-sectional studies in Mali as an example. In doing so, we measured the prevalence of infection in both an urban and rural area of Mali, and estimated the degree to which certain risk factors were correlated with infection in each setting.

## Methods

### Study design

The current study combined data from two cross-sectional studies of HR-HPV in Malian women. Study I recruited urban-dwelling Malian women. The urban study was auxiliary to a study to evaluate the accuracy of visual screening techniques in low-resource settings. A description of that parent study was previously published (see Sankaranarayanan *et al.* 2004 [15]). Another publication assessed the validity of using self-collected cervical samples, and presented a basic analysis of correlates of HPV infection in the urban sample only (see Tracy, JK *et al.* 2011 [9]). Study II (described in more detail in this paper) recruited rural-dwelling women. Both study protocols were approved by the local institutional review committee in Mali and the Institutional Review Board of the University of Maryland School of Medicine.

### Recruitment

Two samples of women were recruited separately: an urban sample from Bamako in March 2007, and a rural sample from the village of Naréna, approximately 100 km (60 mi) southwest of Bamako, in August 2008. The urban sample was population-based, recruited from an age-stratified random sample of women who were selected from a census registry sampling frame. In the rural area, where no census registry existed, women were recruited from the catchment population of a community health center via word-of-mouth advertisement. Participating women in both settings were required to be 15 years or older and provide informed consent. Women under the age of 18 were also required to have verbal consent from a parent or guardian. Women were excluded if they self-reported: (i) history of hysterectomy with removal of the cervix; or (ii) history of anogenital, breast, oral, esophagus, lung, bladder, liver, or cervical cancer.

By local custom, community meetings were held prior to recruitment in both the urban and rural setting. In the urban setting, community recruiters visited the homes of each of the randomly-selected women to invite each to participate in the study. Women who agreed were then assigned appointments at the research clinic, where study personnel explained the study to the women first in groups and then in greater detail individually. An interviewer obtained informed consent in the privacy of an interview room. A printed consent form was translated by a trained investigator into the local language and read to the study participant, and the participant’s signature, index fingerprint, or witnessed verbal affirmation was obtained as indication of consent to participate.

In the rural setting, the study days and times were advertised throughout the community via word-of-mouth. On the study days, potential participants arrived at a community health center, where they underwent an eligibility review and consent process similar to that in the urban setting. The interview was then administered in a semi-private area. Subsequently, the woman entered a private examination room, where a gynecologist performed the pelvic examination and specimen collection.

### Standardized interview

A standardized interview was used to obtain detailed information on sociodemographic factors and sexual and reproductive history. The self-reported value for age should be considered an estimate, as many women, particularly in the rural setting, may not know their exact age. The questionnaire was derived from a more extensive interview used by the International Agency for Research on Cancer (IARC) during an international multisite international study of the epidemiology of cervical cancer [16]. Each interview was conducted prior to cervical screening and specimen collection. A sample questionnaire in English is available (see Additional file 1).

### Clinical examination and specimen collection

Clinical examinations and specimen collection were performed according to standardized procedures [15,17]. A clinician inserted an unlubricated speculum and examined the cervix with the aid of a focus lamp. Cervical cells were collected with a Digene female cervical swab collection kit [15]. Swabs were then placed into a 5-ml vial containing 1 ml of specimen transport medium. Samples were stored at -80°C at a microbiology laboratory in Bamako until shipped to the US for testing.

### HPV DNA testing

HPV DNA testing for samples collected from the urban women was performed in the US using Digene Hybrid Capture II (HC2; Digene, Rockville, MD, USA) according to the manufacturer’s protocol. The HC2 is a pooled-probe, nucleic acid hybridization assay with signal amplification that uses microplate chemiluminescent detection. Each sample was determined to be positive or negative for one of 13 high-risk types of HPV, defined as HPV 16, 18, 31, 33, 35, 39, 45, 51, 52, 56, 58, 59 and 68 [9]. Samples tested by HC2 were considered positive if the viral load was greater than or equal to the mean viral load of the manufacturer-supplied positive control, which contained 1 pg/mL of HPV DNA, corresponding to 5000 or more viral copies. Samples collected from women in the rural group were sent to a different laboratory in the US for HPV DNA testing, which used HC2, Amplicor (using a similar cutoff based on a manufacturer-supplied positive control), and Linear Array (LA)(both Amplicor and LA by Roche Molecular Systems, Pleasanton, CA, USA). Amplicor is a pooled-probe, polymerase-chain reaction (PCR)-based test that targets the same HPV types as HC2, and LA is a type-specific primer PCR assay for 37 HPV types. Specimens testing positive for any of the 13 HR-HPV genotypes by any test or for HPV-66 (because that type strongly cross-reacts via HC2) by LA were considered infected (HR-HPV+).

### Data management and statistical analysis

Interview and clinical examination data were collected using standardized, scannable paper forms, generated by the Teleform™ software system (Cardiff, Sunnyvale, CA, USA).

The variables obtained from the interview were defined as continuous, categorical, or count variables. Average monthly income was reported in West African CFA and was treated as an ordinal variable, with values 1–4 corresponding to increasing income categories.

The urban and rural groups were first compared along sociodemographic and behavioral factors, using Student’s t-tests (continuous and ordinal variables), Pearson’s chi-square tests (categorical variables), Fisher’s exact tests (categorical variables with at least cell of <5 participants), or Poisson regression (counts). We then compared women who were infected (HR-HPV+) to those who were not infected (HR-HPV-), using the same tests. This analysis was then repeated within each geographic setting, with reference categories designated for categorical variables.

To test whether the effects of different variables on HR-HPV infection were different between urban and rural women, we constructed a series of logistic regression models, with HR-HPV status as the outcome variable. Each independent variable was entered into a separate model, along with geographic setting (urban or rural) and a geography×variable interaction term. The significance of the interaction term (or, for variables with more than two categories, the overall significance of the collection of interaction terms) implied how different the effect of the variable was on infection status between the urban and rural women. For categorical variables, this approach produced conclusions that were meaningfully equivalent to those produced by a Breslow-Day test for statistical interaction.

A p-value of <0.05 was considered statistically significant. All analyses were performed using STATA 11 (StataCorp LP, College Station, TX, USA).

## Results

### Participants

The two studies initially recruited a total of 436 participants. HPV testing was not completed in 22 of these women (18 urban women and 4 rural women), and these participants were excluded from the analysis. The final analysis sample included 202 urban and 212 rural women (Table 1), who had a mean age of approximately 34 years in each setting.

**Table 1:** Characteristics of urban and rural samples of women from Mali (n=414)

**Characteristic**	**Urban**		**Rural**		
	**n with data**	**Characteristics**	**n with data**	**Characteristics**	p*
**Total**	202		212		
**Age**	202		210		
Mean (SD)		34.2 (11.9)		33.8 (11.5)	0.72 ^t^
15-24,% of sample within region		23%		24%	0.03
25-34		34%		27%	
35-44		18%		30%	
45-54		15%		14%	
55-65		9%		5%	
**Age menarche,** Mean (SD)	192	14.1 (1.3)	211	14.2 (1.5)	0.22 ^t^
**Can read**	202	29%	211	26%	0.48
**Attended school**	202	38%	211	34%	0.40
**Work outside the home**	195	45%	190	34%	0.03
**Household income/mo.**	191			19%	0.52 ^t^
<25 K CFA (<$50)		26%	196		
25 K-49,999 CFA ($50-100)		33%		47%	
50 K-99,999 CFA ($100-200)		30%		29%	
100 K+ CFA ($200+)		10%		5%	
**Married**	201	69%	207	90%	<0.01
**Polygamous**	183	41%	200	54%	0.01
**Ever pregnant**	201	87%	211	92%	0.08
No. pregnancies, Mean (SD)		4.3 (3.2)		4.9 (3.2)	0.04 ^p^
**No. pregnancies**	200			8%	0.13
0		13%	211		
1-5		51%		47%	
6-8		22%		32%	
9-10		10%		8%	
>11		3%		5%	
**Age first intercourse**	195		209		
Mean (SD)		16.1 (2.5)		15.4 (2.2)	<0.01 ^t^
10-14,%		17%		28%	<0.01
15-18		71%		67%	
19-32		12%		5%	
**Number partners**, Mean (SD)	201	1.6 (1.3)	158	1.8 (1.2)	0.06 ^p^
Regular	201	1.4 (0.8)	158	1.4 (0.7)	0.72 ^p^
Casual	202	0.2 (0.7)	208	0.3 (0.7)	0.04 ^p^
By age 20	202	1.0 (0.7)	209	1.4 (0.8)	<0.01^p^
**Ever used a condom**	191	12%	194	13%	0.69
**Ever had a Pap**	164	3%	149	3%	0.85
**Circumcised**	201	98%	205	97%	0.79

SD: standard deviation.

Twenty-two participants (18 urban and 4 rural) omitted due to missing outcome data. Final analysis dataset included 414 participants.

*Pearson’s chi-square test, except: ^t^Student’s t test and ^p^Poisson regression.

Urban women were not significantly more likely than rural women to be able to read or have ever attended school. More rural than urban women were married and were in polygamous relationships. Rural women also had a marginally higher proportion reporting a prior pregnancy (92% vs. 87%, p=0.08), and reported a greater number of pregnancies (4.9 vs. 4.3, p=0.04).

The rural women appeared to engage in slightly more risky sexual behavior than the urban women. The rural women had first sexual intercourse at a younger mean age than the urban women (15.4 vs. 16.1 years, p<0.01), and had slightly more casual sexual partners and partners before age 20 (1.4 vs. 1.0 mean partners; p<0.01). Few women in either setting used condoms or had ever undergone a Papanicolaou (Pap) test, and nearly all women in both settings were circumcised

### Correlates of HR-HPV infection

Twenty-four (12%) of the urban women had an HR-HPV infection, compared to 49 (23%) rural women (p<0.01; Table 2). The distribution of HR-HPV+ women did not vary significantly by age group.

**Table 2:** Prevalence of HR-HPV infection among subsets of a sample of women from Mali (n=414)

	**Prevalence of HR-HPV**	p*
**Overall**	18% (73/414)	
**Urban**,	12% (24/202)	<0.01
**Rural**	23% (49/212)	
**Age**		
15-24	19% (18/97)	0.96^f^
25-34	19% (23/124)	
35-44	16% (16/101)	
45-54	18% (11/60)	
55-65	13% (4/30)	
**Can read**	20% (23/114)	0.67
Cannot read	17% (50/299)	
**Attended school**	19% (29/149)	0.47
Did not attend school	17% (44/264)	
**Work outside the home**	18% (27/151)	0.68
No	16% (38/234)	
**Household income/mo.**		0.48
<25 K CFA (<$50)	16% (14/88)	
25 K-49,999 CFA ($50-100)	17% (27/155)	
50 K-99,999 CFA ($100-200)	19% (22/114)	
100 K+ CFA ($200+)	20% (6/30)	
**Married**	18% (57/325)	0.71
No	19% (16/83)	
**Polygamous**	17% (32/183)	0.99
No	18% (35/200)	
**Ever pregnant**	18% (67/368)	0.45
No	14% (6/44)	
**No. pregnancies**		0.86^f^
0	14% (6/44)	
1-5	20% (40/202)	
6-8	16% (18/112)	
9-10	15% (6/39)	
>11	19% (4/21)	
**Age first intercourse**		
10-14	26% (24/93)	0.07^f^
15-18	16% (44/277)	
19-32	12% (4/34)	
**Ever used a condom**	20% (10/49)	0.70
No	18% (61/336)	
**Circumcised**	17% (68/395)	0.42^f^
No	27% (3/11)	

HR-HPV: prevalent high-risk HPV; CFA: West Africa CFA franc; $: US dollar.

*Pearson’s chi-square test, except ^f^Fisher’s exact test.

Those who were HR-HPV+ had a somewhat lower mean age at first sexual intercourse compared to HR-HPV- women (15.3 vs. 15.8 years, p=0.08; Table 3). Infected women reported a mean of 1.4 sexual partners by age 20, compared to 1.1 for uninfected women (p<0.01). No other characteristic was meaningfully or statistically different between the HR-HPV+ and HR-HPV- women.

**Table 3:** Characteristics of HR-HPV+ and HR-HPV- women, (n=414)

	**HR-HPV+**	**HR-HPV-**	**p**
**Total,% (n)**	18% (73)	82% (341)	
**Age**, years	33.1 (11.4)	34.2 (11.8)	0.48 ^t^
**Age menarche**	13.9 (1.3)	14.2 (1.4)	0.14 ^t^
**Number pregnancies**	4.6 (3.5)	4.6 (3.2)	0.99 ^p^
**Age first intercourse**	15.3 (2.4)	15.8 (2.3)	0.08 ^t^
**Number partners**			
Total	1.9 (1.5)	1.6 (1.2)	0.13 ^p^
Regular	1.5 (1.0)	1.4 (0.7)	0.08 ^p^
Casual	0.3 (0.9)	0.2 (0.6)	0.31 ^p^
By age 20	1.4 (1.0)	1.1 (0.7)	<0.01 ^p^

Mean (standard deviation).

^t^Student’s *t*-test; ^p^Poisson regression.

### Differences in effects of risk factors between settings

Early sexual intercourse strongly predicted HR-HPV infection among the urban women, but not among the rural women (Table 4). The prevalence of HR-HPV among urban women who had first intercourse from age 10–14 was 26%, compared to 9% among urban women with later sexual debut (p<0.01); such a relationship was not seen among rural women. Condom usage positively predicted HR-HPV infection among urban, but not rural, women; the rate of condom usage in both groups, however, was low.

**Table 4:** Differences in correlates of HR-HPV infection, comparing urban to rural women in Mali (n=414)

	**Urban prevalence**	**Rural prevalence**	p**
**Age**			
15-24	17% (8/47)	20% (10/50)	0.52
25-34	12% (8/68)	27% (15/56)	
35-44	11% (4/37)	19% (12/64)	
45-54	6% (2/31)	31% (9/29)	
55-65	11% (2/19)	18% (2/11)	
**Can read**	14% (8/59)	27% (15/55)	0.90
Cannot read	11% (16/143)	22% (34/156)	
**Attended school**	14% (11/77)	25% (18/72)	0.38
Did not attend school	10% (13/125)	22% (31/139)	
**Work outside home**	11% (10/87)	27% (17/64)	0.45
No	12% (13/108)	20% (25/126)	
**Age first intercourse**			
10-14	26% (9/34)	25% (15/59)	0.07
15-32	9%* (14/138)	22% (33/150)	
**Household income/mo.**			0.28
<25 K CFA (<$50)	6% (3/50)	29% (11/38)	
25 K-49,999 CFA ($50-100)	13% (8/63)	21% (19/92)	
50 K-99,999 CFA ($100-200)	10% (6/58)	29% (16/56)	
100 K+ CFA ($200+)	20% (4/20)	20% (2/10)	
**Married**	9% (12/139)	24% (45/186)	0.09
No	19%* (12/62)	19% (4/21)	
**Polygamy**	9% (7/75)	24% (25/108)	0.69
No	12% (13/108)	23% (22/92)	
**Ever pregnant**	12% (21/174)	24% (46/194)	0.76
No	11% (3/27)	18% (3/17)	
**No. pregnancies**			0.53
0	11% (3/27)	18% (3/17)	
1-5	17% (17/102)	23% (23/100)	
6-8	7% (3/45)	22% (15/67)	
9-10	5% (1/21)	28% (5/18)	
>11	0% (0/7)	30% (3/10)	
**Ever used condom**	26% (6/23)	15% (4/26)	0.03
No	11%* (18/168)	26% (43/168)	

HR-HPV: high-risk HPV.

*Within residence setting, significantly different from first listed category, using Pearson’s chi-square test.

** p-value for difference in relationship between characteristic and HR-HPV status between areas, using logistic regression.

The lifetime number of pregnancies was negatively correlated with HR-HPV infection among urban women, with infected women reporting 1.7 fewer mean pregnancies than did uninfected women (p<0.01), but no significant difference was seen among rural women (Table 5). Infected urban women reported a mean of 1.3 sexual partners by age 20, compared to 0.9 for the uninfected women (p=0.01). However, age at first intercourse and numbers of sexual partners were not predictors of infection status among rural women.

**Table 5:** Characteristics of HR-HPV+ and HR-HPV- women, by region (n=414)

**Characteristic**	**Urban**		**Rural**		p**
	**HR-HPV+**	**HR-HPV-**	**HR-HPV+**	**HR-HPV-**	
**Age**, years	31.2 (12.0)	34.6 (11.9)	34.1 (11.1)	33.7 (11.6)	0.23
**Age menarche**	13.7 (1.3)	14.1 (1.3)	14.0 (1.3)	14.3 (1.5)	0.68
**Number pregnancies**	2.8 (2.4)	4.5 (3.3)*^p^	5.5 (3.6)	4.8 (3.1)	0.01
**Age first intercourse**	14.9 (1.9)	16.3 (2.5)* ^t^	15.5 (2.6)	15.4 (2.1)	0.01
**Number partners**					
Total	2.0 (2.0)	1.5 (1.2)	1.9 (1.1)	1.8 (1.2)	0.44
Regular	1.6 (1.0)	1.4 (0.8)	1.5 (1.0)	1.4 (0.6)	0.14
Casual	0.4 (1.2)	0.1 (0.6)	0.3 (0.6)	0.4 (0.7)	0.12
By age 20	1.3 (0.9)	0.9 (0.7)* ^p^	1.5 (1.0)	1.3 (0.7)	0.16

Mean (standard deviation).

HR-HPV+: tested positive for high-risk HPV; HR-HPV-: tested negative for high-risk HPV type.

*Significant difference (p<0.05) within area, using ^t^Student’s *t* test; ^p^Poisson regression.

**p-value for difference in relationship between characteristic and HR-HPV status between areas, using logistic regression.

## Discussion

The study demonstrated that the occurrence and patterns of HPV infection may be different between urban and rural settings within the same developing country, using data from Mali as an example. Our sample of rural women had nearly twice the prevalence of HR-HPV as did an urban sample. Early sexual intercourse and fewer lifetime pregnancies predicted HR-HPV infection, but only in urban women.

### Geographic setting

The much higher prevalence of HR-HPV in rural compared to urban women is not easily explained by sampling techniques, despite differences in recruiting methods that were used in the urban and rural settings of this study. In this and previous studies in these settings, we have encountered a population that is generally very receptive of medical research studies and is eager to participate, regardless of current or previous medical conditions or experiences. The sampling techniques produced samples that were comparable on many sociodemographic characteristics, providing evidence that the women who decided to participate were similar across the settings. The baseline differences that were apparent-for example, the large number of 35–44 year-old rural women, which was likely due to the estimation of ages in the rural setting-did not appear to bias the overall prevalence figures within settings, as demonstrated by subgroup prevalence figures. We therefore do not expect that differences in recruiting techniques, alone, could explain the wide disparity in prevalence between the two settings, nor could it solely explain the most apparent differences in HPV patterns.

The difference in prevalence between settings is also unlikely to be caused solely by behavioral or sociodemographic predispositions. The idea that the high rural prevalence is driven by that group’s riskier sexual behavior-a somewhat lower mean age at first intercourse and slightly higher number of partners before age 20-is refuted by the lack of a convincing association between either of those risk factors and HR-HPV status in the rural setting. Several possible explanations remain regarding difference in prevalence between settings; for example, an historic founder effect could contribute to the results. The prospect also exists that sexual and social network construction is inherently different in the rural setting, perhaps due cultural factors such as the occurrence of polygamy, in such a way that facilitates HPV transmission to nearly all women quickly upon sexual debut.

### Number of pregnancies

Contrary to the Bayo *et al.* study-a hospital-based case-control study of cervical cancer from the mid-1990s in Bamako [12]-this study failed to find a positive relationship between parity and HR-HPV. In fact, among our urban sample, infected women reported fewer lifetime pregnancies than did uninfected women. In the Bayo *et al.* study, women with six or more pregnancies had at least three times the odds of invasive cervical cancer compared to those with five or fewer pregnancies. Historically, studies in the developing world-but not in developed countries-have found a positive relationship between parity and HPV or cervical cancer [3,12,18,19]. This phenomenon suggests that the measured relationships with parity may be the result of residual confounding due to sexual activity, and can be eliminated in populations with high use of contraceptives. Our finding of a negative relationship between parity and HR-HPV in the urban group may reflect changing sexual dynamics. It is possible that our recruiting strategy may have yielded a small number of sexually active urban women who were unlikely to marry or have many children, a group that may have either been rare or otherwise not detected previously. The association between condom usage and HR-HPV infection in urban women seems to support this idea. Likewise, the association between high parity and HR-HPV infection may be eroding in rural parts of West Africa. In a study in rural southern Nigeria published in 2011, prevalence of HR-HPV was virtually identical across all parity groups [20], as it was in our rural sample.

### Other risk factors

Early sexual debut was a risk factor of HR-HPV infection in our urban sample, a result that has been found elsewhere in the developing world [3,4], but could not be detected at a significant level by previous studies in Mali [9,12]. This finding should not be read to suggest that delaying sexual debut would be effective at reducing the burden of cervical cancer in this population, or even possible in a society with strict gender expectations. Instead, the larger picture of HPV and sexual risk factors depicted by this study implies that many traditional proxies for risky sexual behavior found elsewhere in the world do not strictly apply in the context of Mali.

For some factors, such as female circumcision, lack of screening, and lack of screening knowledge, the sample was too homogenous to detect any meaningful association with HR-HPV status.

### Effect of high prevalence on research and planning

The high baseline prevalence of HPV in this population may obscure the effect of risk factors measured through a cross-sectional study. Beyond the detectable group of HPV-infected women was a presumably much larger but undetectable (by this study) group of women who had recovered from an HPV infection. The HPV prevalence rates seen here were comparable to those measured elsewhere in West Africa [21]. In areas where nearly one in four (rural) or one in eight (urban) women is infected at any given time, nearly all women may expect to be infected with an HR-HPV type-often multiple times-in their lifetimes. In the Bayo *et al.* study, 60% of women tested positive for having ever been infected with the three most common HR-HPV types, and nearly all women had a previous infection with any HPV type [12]. Because of the high occurrence of the virus, research using snap-shot glimpses of prevalence may not be able to distinguish between women who engage in high-risk behaviors and those who do not, because the infected group may be a seemingly random selection of those who will ever be infected-a group which contains most of the female population. The high prevalence of HR-HPV in the rural setting would make it particularly difficult to find meaningful associations there.

Because HR-HPV infection may be nearly inevitable for many women in Mali and other parts of the developing world, even in the absence of high-risk behaviors, the focus of vaccination efforts in such resource-limited settings should be on the general female adolescent populations of highly-affected areas, like our rural setting. Based on our results, behavioral intervention may be unlikely to stem the course of HPV infection and cervical cancer. Furthermore, seeking out potential high-risk members (based on projected future behavior or sociodemographic factors other than age) within populations may add an unnecessary layer of difficulty and cost to vaccine implementation. Vaccination in general populations will likely be effective when implemented; a recent mathematical modeling simulation of the Malian population projected that the HPV vaccine could reduce the burden of cervical cancer by approximately the same proportion as vaccine coverage rates [22].

### Limitations and future directions

This study used different laboratory testing methods to detect HR-HPV infection in the urban and rural settings-HC2 alone was used in the urban setting, whereas the rural setting saw HC2, LA, and Amplicor. However, any overestimation in HPV prevalence in the rural setting due to the use of parallel tests is expected to be modest. Previous work in a cohort of women with cervical abnormalities in Australia has shown generally high agreement between the three tests, with interassay agreement ranging of about 84% comparing either Amplicor or LA to HC2, and 98% comparing Amplicor to LA [23]. Agreement between the three tests was shown to be similarly high in the Mississippi Delta region of the United States [24]. Despite our study’s setting in low-resource areas, we were able to perform sample collection and maintenance according to Western standards with well-trained clinical personnel who had previous research experience, and we analyzed the samples in university laboratories in the United States; we therefore do not anticipate that difficult analytic or pre-analytic conditions could have contributed meaningfully to the test results. For these reasons, we are comfortable asserting that that the difference we found in prevalence between regions could not be solely an artifact of different testing methods.

Interpretation of these findings should be careful to consider the number of simultaneous statistical comparisons being made. Also, the statistical power of the study to identify risk factors was limited both by the high prevalence of HR-HPV, especially in the rural group, and the number of people in each setting.

As with all studies using self-reporting of variables, especially variables of a sensitive sexual nature, the quality of our data is contingent on accurate reporting. Misclassification of sexual behavior variables likely would have made it more difficult for us to find significant differences between urban and rural or infected and uninfected women. The main research variables-HR-HPV status and geographic setting-were not prone to misclassification due to self-report.

The finding that HPV occurs according to different patterns in urban and rural settings could have implications that reach far beyond West Africa. Because associations that appear obvious in one area may not be present in others, planners of vaccination campaigns must do exploratory work in every potential setting to measure the burden of disease and uncover patterns of HPV and cervical cancer occurrence.

## Conclusions

As HPV vaccine implementation unfolds in the developing world, public health planners within countries must identify the subpopulations with the greatest need, so that they can prioritize the allocation of limited resources. This study demonstrated that data from urban areas-where most cervical cancer and HPV surveillance has been done-cannot be used to predict HPV occurrence in rural areas. We tested more than 200 women for prevalent HR-HPV infection in each an urban and rural setting in Mali, West Africa. HR-HPV infection was twice as prevalent among our rural sample of women compared to our urban sample. Earlier sexual debut and fewer pregnancies predicted HR-HPV infection among urban but not rural women. In areas with very high prevalence of HPV, the practice of identifying risk factors can be troublesome from statistical and practical perspectives. The ubiquity of HPV in such a setting may erode the apparent effect of risk factors other than geography, meaning that to vaccination planners, nearly all adolescents in high-burden areas can be considered at high risk for contracting HPV and developing cervical cancer.

## Abbreviations

HPV: Human papillomavirus; HR-HPV: High-risk human papillomavirus; HC2: Hybrid Capture II; LA: Linear Array; PCR: Polymerase chain reaction

## Competing interests

The authors declare that they have no competing interests.

## Authors’ contributions

KT and NS led the writing of the manuscript and the data analysis. In addition, KT conceptualized the study design and methods and secured funding for these studies. SS led the organization and implementation of the study in Mali. CT, KB, RD, and FS helped to design the recruitment and data collection procedures. PG was involved with the conception of the study design and methods and performed HPV testing of rural samples. All authors had access to the data, and have approved of the data analysis and the manuscript. All authors read and approved the final manuscript.

## Pre-publication history

The pre-publication history for this paper can be accessed here:

http://www.biomedcentral.com/1472-6874/13/4/prepub

## Supplementary Material

Additional file 1 **Mali Interview-English.** Description: English version of the questionnaire administered to both urban and rural participants. Click here for file
