# Supplementary material for: Differences in patterns of high-risk human papillomavirus infection between urban and rural low-resource settings: cross-sectional findings from Mali
Source: BMC Womens Health. 2013 Feb 6;13:4. doi: 10.1186/1472-6874-13-4 (PMC3599375; doi:10.1186/1472-6874-13-4)
Supplement: Additional file 1 — Mali Interview-English. Description: English version of the questionnaire administered to both urban and rural participants. [file 1472-6874-13-4-S1.pdf]

Questionnaire for Pilot Study of Factors Associated with HPV Infection in Malian Women

Pilot Study of HPV
Questionnaire

Good morning (or afternoon).

My name is ....and first of all I would like to thank you for having accepted to participate in this study. We are conducting a study in Mali to clarify if certain characteristics and habits of men and women are related to the virus that causes cervical cancer. For this purpose we will interview many women attending this clinic or other hospitals.

If you agree I will ask you several questions and the answers will be recorded on this form.

I would like to reassure you that all that is said during the interview will be strictly confidential and that the information collected will only be used in scientific reports without any personal name or identifiers being mentioned.

Any likely benefits of the study for the well being of Malian women rely on the accuracy of your answers. Therefore, if you do not understand the meaning of any of the questions, please don't be afraid to ask.

At any time you may refuse to continue or to answer specific questions. In addition to the interview, the study includes a Pap smear and a blood sample.

Can we start now?

A. Personal Data

1. Quartier

2. Sub-quartier

3. How many years have you lived there?

4. Ethnic group

5. What is your approximate age?

6. Can you write?

7. Can you read?

8. Have you ever gone to school?

9. What was the highest level of education you reached?
   --primary incomplete
   --primary complete
   --secondary incomplete
   --secondary complete
   --college
   --technical school
   --university

10. How old were you when you left school?

11. Have you ever worked outside the home?
 --yes
 --no

12. Were you?
 --employed
 --self-employed
 --manager
 --supervisor
 --employer
 --other (specify)

13. What sector did you work in?
 --agriculture/farming/fishing
 --textile industry/manufacturing
 --industrial/manufacturing other than textile
 --administration/office
 --services and sales
 --domestic (other house)
 --other (specific)

14. Average monthly income:

---24.999 cfa

--25.000-49.999 cfa

--50.000-99.999 cfa

--100.000-199.999 cfa

--+200.000 cfa

15. Marital status
 --married
 --single
 --divorced
 --separated

--widow

--cohabit

16. At what age did you marry for the first time?

17. How many times in your life have you married?

18. Do you or have you ever smoked?
 --yes
 --no

19. How old were you when you started to smoke regularly?

20. Do you still smoke?
 --yes
 --no

 21. If no, how old were you when you stopped smoking?

B. Reproductive History

Now, let me ask you about your reproductive life.

1. How old were you when you started menstruating/your period?

2. Are you still having periods?
   --yes
   --no

3. How old were you when they stopped completely?

4. Have you ever been pregnant?
   --yes
   --no

   [IF NO, go to C1]

5.How many pregnancies have you had?

6. What type(s) of deliveries have you had?
   --vaginal
   --vaginal with episiothomy
   --vaginal with forceps
   --caesarian

7. Where did you deliver your children? [check all that apply]
   Home (yes/no)
   Hospital (yes/no)
   Private clinic/private hospital (yes/no)
   Health center (yes/no)
   Other (yes/no)
      If no, specify

8. Did you have any bad consequences due to the birth [what kinds of problems initiated within 40 days after the delivery?
   None (yes/no) [IF NO, go to B11]
   Urinary problems—incontinence, leakage, infections (yes/no)
   Uterine problems—prolapse, sterility (yes/no)
   Pelvic or other general health problems—fistulae, infections of the pelvis, fever, abdominal pain, lochia (yes/no)
   Don’t know (yes/no)

9. For how long did you suffer from this complication/condition?
   --less than one month
   --1-6 months
   --more than 6 months
   --don’t know

10. For how many of these pregnancies did you have antenatal care—at least one visit with a physician, a nurse, or a trained midwife before giving birth?

Now, we would like to ask you some questions about your sexual behavior during the periods you were pregnant. We know that these are very personal questions, but they are necessary to complete the history of your pregnancies.

11. Have you ever had sexual intercourse while being pregnant?
   --no, never when pregnant [IF NO, go to B14]
   --yes, rarely

--yes,. regularly

12. Did you have sexual intercourse while being pregnant in…
 --all your pregnancies
 --more than 50% of your pregnancies
 --less than 50% of your pregnancies

13. During the pregnancies in which continued to have sexual intercourse, up to what time did you usually have intercourse?
 --up to 1-3 months
 --4-6 months
 --7-9 months

14. On average, after your pregnancies, how soon after delivery/pregnancy termination did you start having sexual intercourse?
 --immediately
 --1st – 3rd month
 --4th – 6th month
 → 6th month

15. Have you ever used a condom?
 --yes
 --no

16. What was it made of (local terms)?
 --commercial rubber
 --other

17. How many years have you used it?

C.**Sexual history**

In this part of the questionnaire we would like to ask you about your sexual life with your partners. We are quite aware that these questions are very personal and sometimes you may hesitate in answering them. We know that sexual habits have an influence on people's health. Therefore we hope that your answers will be as complete and truthful as possible.

1. How old were you when you had sexual intercourse for the first time?

2. How old were you when you began to have intercourse at least once a month?

 [IF FIRST INTERCOURSE BEFORE 20 YEARS OLD]

3. How many partners did you have before 20 years of age?

Now, I would like to ask you about any regular sexual partner/husbands you have had through your life. [*Regular partner is a person with whom you have had a regular sexual relationship for at least 6 months with or without a legal or religious contract of marriage, irrespective of living in the same house or not.

4. How many regular sexual partners have you had in your life?

5. How many sporadic/casual partners have you had in your life

6. While with this partner did you use any contraceptive method?
 --yes
 --no
 --don't know

7. IF YES: Which contraceptive methods did you use?
--rhythm/withdrawal (yes/no)
--condom (yes/no)
--diaphragm (yes/no)
--oral, injectable, or implant (yes/no)
--IUD (yes/no)
--foam/spermicides (yes/no)
--“tubes tied” or hysterectomy (yes/no)
--vasectomy (yes/no)
--other (yes/no)—specify

D. Pap smear history

1. Do you know what a Pap smear is?
--yes
--no

1a. IF YES: record participant’s description.
Interviewer codes description:
--Yes, she knows what a Pap smear is
--She has some idea but is uncertain if she could distinguish it from other medical procedures.

2. Do you think a Pap smear can help detect cancer earlier?
--yes
--no
--don’t know

3. As far as you know, have you ever had a Pap smear?
--yes
--no
--don’t know

[IF NO, skip to E1]

4. At what age was your first Pap smear?

5. Since your first Pap smear, how often have you had others taken?
--yearly
--once every 2-3 years
--once every 4-5 years
--once every 6-10 years
--less than every 10 years

6. Approximately how many Pap smears have you had in your life?

7. Where did you get your Pap smear(s)?
--home (yes/no)
--health center (yes/no)
--hospital (yes/no)
--private clinic/doctor’s office (yes/no)
--other (yes/no)-specify

8. Who performed the smear(s)?
--general practitioner (yes/no)
--cytopathologist (yes/no)
--gynecologist (yes/no)
--nurse/midwife (yes/no)
--other (yes/no)-specify

9. Were you referred to hospital or to perform another exam as a result of any Pap smear?
--yes
--no
--yes, but didn't go
--don't know

10. Did you receive any of the following treatments:
--creams/liquids in the genitals (yes/no)
--small surgery on the cervix/conization (yes/no)
--electrocauterization of the cervix (yes/no)
--laser or cryosurgery of the cervix (yes/no)
--uterus removed (hysterectomy) (yes/no)
--radiotherapy (yes/no)
--other (yes/no)—specify

E. Repeat of Mali-specific questions from previous IARC study

1. Have you ever been one of the wives in a polygamous family/relationship (the same husband with more than one wife)
--yes
--no

2. Besides yourself, how many women were married to your husband at the same time that you were?
--1
--2
--3
--4 or more
--don't know

3. Are/were all the wives living in the same house as you?
--yes
--no
--don't know

5. Do you know what female circumcision is?
--yes
--no

6. Have you ever been circumcised?
--yes
--no
--don't know

7. Could you tell me at approximately what age you were circumcised?

8. Who did the circumcision?
--midwife
--elderly woman
--witch doctor
--nurse
--doctor
--other (specify)
